# Convergence model for effectual prevention and control of zoonotic diseases: a health system study on ‘One Health’ approach in Ahmedabad, India

**DOI:** 10.1186/s12961-018-0398-6

**Published:** 2018-12-19

**Authors:** Sandul Yasobant, Walter Bruchhausen, Deepak Saxena, Timo Falkenberg

**Affiliations:** 1 Center for Development Research (ZEF), University of Bonn, Bonn, Germany; 2 University of Cologne, Köln, Germany; 3 Indian Institute of Public Health Gandhinagar, Gujarat, India

**Keywords:** Systems thinking, prevention and control, zoonotic diseases, One Health, health systems

## Abstract

The complexity and increasing burden of zoonotic diseases create challenges for the health systems of developing nations. Public health systems must therefore be prepared to face existing and future disease threats at the human–animal interface. The key for this is coordinated action between the human and the animal health systems. Although some studies deal with the question of how these two systems interact during unforeseen circumstances such as outbreaks, a dearth of literature exists on how these systems interact on early detection, prevention and control of zoonotic diseases; assessing this problem from the health system perspective in a developing nation adds further complexity. Systems thinking is one of the promising approaches in understanding the factors that influence the system’s complexity and dynamics of health maintenance. Therefore, this study aims to understand the generic structure and complexity of interaction between these actors within the domain of One Health for the effectual prevention and control of zoonotic diseases in India.

The present study will be executed in Ahmedabad, located on the Western part of India, in Gujarat state, using a mixed methods approach. For the first step, zoonotic diseases will be prioritised for the local context through semi-quantitative tools. Secondly, utilising semi-structured interviews, stakeholders from the human and animal health systems will be identified and ranked. Thirdly, the identified stakeholders will be questioned regarding the current strength of interactions at various levels of the health system (i.e. managerial, provider and community level) through a quantitative network survey. Fourthly, utilising a vignette method, the ideal convergence strategies will be documented and validated through policy Delphi techniques. Finally, through a participatory workshop, the factors that influence convergence for the control and prevention of zoonotic diseases will be captured.

This study will provide a comprehensive picture of the current strength of collaboration and network depth at various levels of the health system. Further, it will assist different actors in identifying the relevance of possible One Health entry points for participation, i.e. it will not only contribute but will also develop a system convergence model for the effectual prevention and control of zoonotic diseases.

## Introduction

The research literature cites an increasing burden of emerging, re-emerging and endemic zoonotic diseases that are attributed to complex linkages at the human–animal–ecosystem interfaces [1, 2]. The One Health approach, which recognizes that the health of people is connected to the health of animals and the environment, is the most appropriate approach for the sustainable management of zoonotic diseases [3], as well as for their prevention and control [4–6]. At both the national and global levels, an increasing trend can be witnessed towards One Health approaches in order to tackle the challenges of zoonotic diseases in the most effective way [7–9]. Various challenges, such as the complex nature of zoonotic diseases as well as the limited resources of developing countries, make implementation of the One Health approach more crucial [2]. As the One Health approach focuses on collaboration with various stakeholders, its implementation represents a complex process for health systems, especially for those with feeble structures in developing nations [10–12].

The operationalisation of the One Health approach endures challenges in both developing and developed nations [13–15] due to the lack of a shared vision and culture, which should be more collaborative and accommodative of all sectors concerned with the human–animal interface in health. Furthermore, this approach can only be successfully operated if backed by enabling governance structures with clearly defined roles and responsibilities for each sector [15, 16]. Available evidence also indicates that the collaborative efforts between physicians and veterinarians in communication, sharing of public health knowledge and research settings could do much in managing and controlling zoonoses [17–19].

Our literature review indicates three different types of collaboration and partnerships for implementing One Health. The first type is ‘solution-based’ collaboration [7], i.e. joint outbreak management or planned integrated health services like the case of the Chad joint immunisation programme [20]. Here, solving a defined problem, e.g. difficulties in controlling an acute epidemic or in reaching remote populations for preventive interventions, is the starting point for joint action between human and animal health services. The second type is ‘third-party based’ collaboration [21], i.e. establishing a third party that can act as a knowledgeable or trusted intermediary between the stakeholders, for example, the strategic framework of the Bangladeshi One Health secretariat [22]. The third type is the most sustainable kind of collaboration, based on respective level (individual level, population level or research level) collaboration [10]. Establishing such a ‘level oriented’ collaboration requires a profound understanding of the complexity of the respective health systems, especially in a country like India, with its lack of existing or effective mechanisms to bring together the stakeholders who need to be involved in zoonoses research or control management [23–25].

To build resilience in the health system, efficient resource allocation is vital [26]. Systems thinking has been tested and proven a successful approach for understanding the complexity and dynamics of health networks [27–30]. General systems theory is also anchored in the One Health approach [31]. Essentially, systems thinking is an approach to problem solving and designing solutions, where the role and mutual influence of stakeholders and context is unclear [28, 32, 33]. With an axiomatic approach, systems thinking can complement the linear and reductionist approaches by permitting the testing of new ideas in social systems [29]. In systems thinking, an organisation and its respective environment (context) are viewed as an entangled whole of interrelated and interdependent parts rather than separate entities [29, 34]. This takes into account the structures, patterns of interaction, events and organisational dynamics as components of larger structures, helping to anticipate rather than react to events, and to prepare better for emerging challenges.

Therefore, this study aims to understand the generic structure and convolutedness of interaction between the various sections of the human and animal health systems within the domain of One Health for an effective prevention and control of zoonotic diseases in India. More specifically, it aims to build an understanding of how the various sections within the human and animal health systems are currently interacting. Further, the study will attempt to document the factors facilitating or hampering the development of effective convergence between these two health systems in Ahmedabad, India.

The specific research objectives are:

1. To identify the major zoonotic diseases of public health importance in Ahmedabad city
2. To identify and categorise the stakeholders within the human and animal health systems responsible for prevention and control of zoonotic diseases in Ahmedabad city
3. To examine the current strength of collaboration between the identified stakeholders at various levels of the health system
4. To develop new convergence strategies for effective prevention and control of zoonotic diseases
5. To document the factors that influence enhancing convergence between the human and animal health systems

## Methods

### Study design

This study entails a mixed methods approach consisting of both quantitative and qualitative data collection (interviews, survey and participatory workshops).

## Study setting

This study will be implemented in the city of Ahmedabad. It is the seventh most populous city in India and the largest city of the Western state of Gujarat, India [35]. It is located on the banks of the Sabarmati River with a population of 7,650,000 [36].

The Union Ministry of Health and Family Welfare at central level governs human health in India. In each State, there is a State Department of Health and Family Welfare that is headed by a State Minister and a Secretariat under the charge of the Secretary/Commissioner (Health and Family Welfare). The Indian health system consists of both allopathy and AYUSH (Ayurveda, Yoga, Unani, Siddha and Homeopathy). There is a three-tier system, wherein the primary level includes village teams, sub-centres and primary health centres, the secondary level is composed of community health centres and sub-district hospitals, and the tertiary level consisting of district hospitals and medical colleges to provide rural healthcare. In contrast, the urban health system relies upon urban health centres and medical colleges [37]. Animal health is one of the subjects of the Department of Animal Husbandry, Dairying and Fisheries under the Ministry of Agriculture. In all districts there are Offices of Deputy Director of Animal Husbandry or Assistant Director of Animal Husbandry, directing veterinary dispensaries, branch veterinary dispensaries, mobile veterinary dispensaries, first aid veterinary centres, etc.

Specifically in Ahmedabad, human health services are controlled by two different governance systems, i.e. urban health governed by the Department of Health at Ahmedabad Municipal Corporation and rural health governed by District Panchayat of Ahmedabad district. The rural areas of Ahmedabad have one district hospital, six community health offices and 36 primary health centres [38], whereas the urban areas of Ahmedabad have six urban health centres, six medical colleges and one homeopathy college as well as being well facilitated by private companies for human health [35]. Similarly, animal health is controlled by the Cattle and Nuisance Control Department under Ahmedabad Municipal Corporation for urban areas and the Department of Animal Husbandry under District Panchayat for rural Ahmedabad. There are 26 veterinary hospitals and 17 primary animal treatment centres, which are available throughout the rural part of Ahmedabad [38] compared to only four veterinary dispensaries across the city. Healthcare provision by trusts (non-profit agencies) and profitable private sector facilities are also available widely to contribute towards animal healthcare in the city.

### Research design

**Fig. 1:**
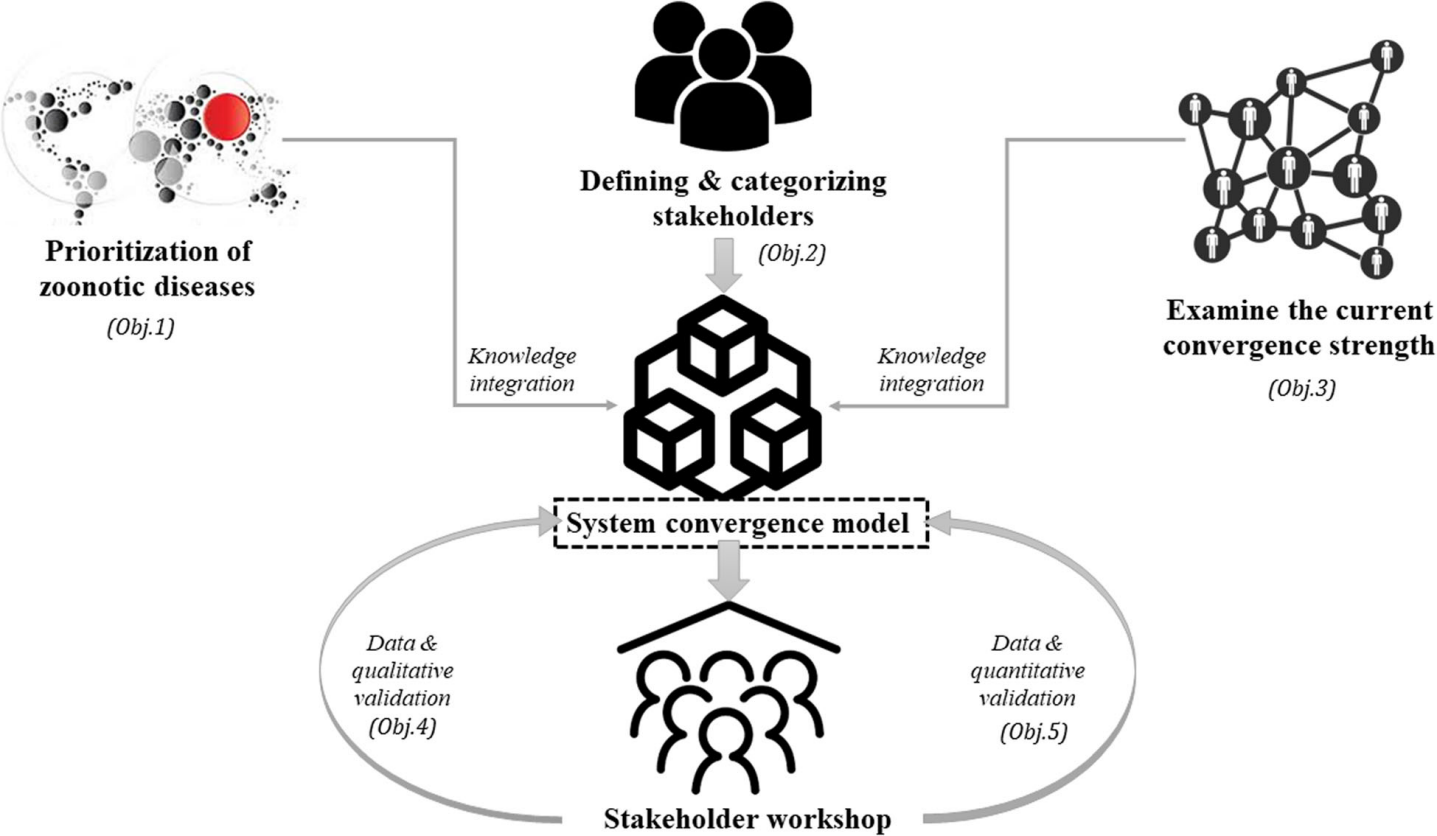
Analytical framework of the Research to explore Intersectoral Collaborations for the One Health Approach (RICOHA) study

The analytical framework (Fig. 1) illustrates the research design of the study. The study will begin with prioritisation of zoonotic diseases of public health importance in Ahmedabad city (Objective 1). The system exploration will commence by defining and categorising the stakeholders in order to understand the influence of the various actors in the health system(s) (Objective 2). This will be followed by assessing the strength of the current interaction and the collaboration strategies through a network survey (Objective 3). After having analysed the system actors and their current level of interaction, the possible ways to further develop the systemic interaction will then be analysed through a vignette approach, which will be validated through the policy Delphi method (Objective 4). Finally, based on the consensus documented throughout the previous phases, the factors essential for developing the convergence will be captured through a participatory workshop. A sensitivity analysis will be conducted to conclude the important factors for developing convergence in relation to the local health system (Objective 5). The One Health entry points, which will have been explored during the previous objectives (Objectives 1–3), will be validated (Objectives 4 and 5) further through qualitative (vignette) and quantitative (sensitivity analysis) approaches.

The system convergence model from this study will be a qualitative system model, generally used to explain the system’s internal feedback loops to make its relationships easier to understand. This approach has also been successfully employed to enhance the development of health policies and programmes [39, 40].

### Sample and sampling strategy

As this study consists of harmonised objectives, information from previous objectives is required to proceed to the next objective. This study will draw samples from three different strata of the health system structure, i.e. from the managerial/decision-making level, from the service provider level and from the community.

- **For the managerial level:** The sampling unit for this category will be individual actors in managerial positions in either the human health or animal health system or other related environmental programmes at the city level. The purposive sampling strategy will be adapted to recruit subjects from this category. This category involves the following types of actors:
  ■ *Managerial actors:* Individuals working as managers, programme officers or decision-makers, involved in the planning of human health services at the Ahmedabad Municipal Corporation or at the Animal Husbandry Department for animal health services.
  ■ *Surveillance actors*: Individuals such as epidemiologists, entomologists, statistician or managers, working in the surveillance system, i.e. Integrated Disease Surveillance Project for human health or National Animal Disease Reporting System for animal health.
- **For the service provider level:** The sampling unit for this category will be individual actors from both the human and the animal health systems who are involved in delivering health services directly or indirectly. Both the public and private sector actors will be considered for this category. The snowball sampling strategy will be applied to recruit actors within this category, as no complete list of private service providers is available. This category involves the following types of actors:
  ■ *Clinicians:* Physicians who are involved in managing infectious diseases or veterinarians providing animal healthcare.
  ■ *Laboratories:* The laboratories that are involved in conducting tests on human or animal samples for zoonotic diseases.
  ■ *Professional bodies:* The professional bodies such as the Indian Medical Association, Gujarat branch, and the Gujarat Veterinary Association will belong to the key actors under this category.
- **For the community level:** The sampling unit for this category will be those individuals who have contact to both the human and the animal health systems, i.e. from households having any domestic animals (either for profit or for non-profit). The person responsible for taking care of the animals will be the interviewee for this category. In addition to this, directors of non-governmental organisations working in the community related to zoonotic diseases will be included under this category. The simple random sampling will be adapted to select the households that have contact to both systems. Initially, a list of households affected by the last zoonotic outbreaks will be obtained and then selection will be done randomly to recruit for this study.
- **Additional sample:** An additional sample of experts will be recruited for objective 4. Experts from academia, research, government, international/national agencies, etc. will be approached purposively.

### Method for objective 1

Joint prioritisation of zoonotic diseases has the potential to benefit both the human and the animal health systems, especially in resource-scarce settings. It might be of help for comprehensive planning to conduct efficient and effective surveillance, develop laboratory capacity, target outbreak response and implement disease control strategies. However, prioritisation of zoonotic diseases is more important where there is a paucity of quantitative data for decision-making. Taking a collaborative approach to the priority-setting process ensures equal input from stakeholders in both human and animal health sectors, and ideally results in a ranked list of zoonoses that can inform joint efforts in areas of overlapping interest. Prioritisation of zoonoses is becoming an integral step for initiating One Health collaboration and is being implemented in both developed [41] and developing nations [42]. The specific purpose of this joint prioritisation within the study is to rank the zoonotic diseases that are especially important for Ahmedabad city. Purposive sampling is proposed to recruit 10–12 stakeholders from the managerial level (i.e. both managerial and surveillance actors). A participatory workshop is planned for this objective and the guidelines from the United States Centers for Disease Control and Prevention will be followed [43].

To prioritise zoonotic diseases in the city, a semi-quantitative tool, i.e. the One Health Zoonotic Disease Prioritisation tool developed by Rist et al. [43], will be adapted for this local setting. Prior to the administration of this tool, a literature review will be conducted to collect secondary information on zoonotic diseases concerning India and Gujarat, including outbreak information from the last 5 years. This tool will be administered in five steps, either through individual or group work, consisting of listing of zoonotic diseases, deciding the criteria for weighing, developing the questions under each criteria, ranking the criteria and ranking the diseases based on the criteria. These data from the workshop is planned to be analysed with help of the Analytical Hierarchy Process [44] and decision tree analysis to highlight the top prioritised diseases [43].

### Method for objective 2

Stakeholder identification is an important step for understanding the diverse actors for prevention and control of zoonotic diseases within the human and the animal health systems. Stakeholder identification is an iterative process in health system research that provides better insights into system complexity regarding roles and engagement [45, 46]. This method is used extensively in various fields of social science, e.g. identifying stakeholders for a specific project [47, 48].

The sample for this objective will be recruited from the managerial level (i.e. both managerial and surveillance actors) and from the service provider level. Approximately 10–12 key influential actors from both the systems will be recruited for this objective or until the saturation of responses. Semi-structured interviews will be conducted with the sampled actors. If, during the interviews, any new actors are identified, then they will be added to the stakeholders list and considered for further interviews. To understand their influence at different levels of the health system (managerial, providers, community), a quantitative ranking of actors will be applied. The ranking scale is based on the response to a question about ‘high-medium-low’ influence, which will be asked to each participant in order to rank other actors during the interview. In addition, the type of collaboration exercised by these stakeholders will also be documented.

Transcripts will be made the same day based on the verbatim notes from the interview. Both inductive and deductive codes will be generated; similar codes will be combined into themes [49]. To ensure that the results are a reflection of the data, the codes/themes will be related back to the original data [50]. The qualitative data will be reported by using the Consolidated Criteria for Reporting Qualitative Research [51] after analysing through ATLAS.ti version 8 [52]. The stakeholder analysis will be conducted based on the Hyder model [53]. The final stakeholder analysis is performed as a stakeholder metric emphasising the Interest and Influence Matrix [54, 55], which is usually implemented in workshops. However, power relationships during workshops could hinder the assessment process in the study area, so we preferred interviews to allow respondents to assess the other actors confidentially [56].

### Method for objective 3

To examine the strength and pattern of the current convergence between the actors, a network survey is planned. Network surveys have been extensively used not only in public health research [57–59], but also in health systems research [60].

All three sample categories will be applied for this objective. To recruit samples under this objective, the purposive sampling for the actors from the managerial level, the snowball sampling for the actors from the providers’ level and the simple random sampling for the households will be adapted.

A structured network questionnaire will be administered to each participant. This structured network questionnaire will differ between stakeholder categories, as the actors have different roles within the system. Here, we are interested in examining the complete networks, i.e. all actors, including public and private actors from the human and animal health system. We will be applying both types of choices, namely stakeholders are chosen from a given list or by free calling, i.e. stakeholders are chosen unrestrictedly, to document the interaction with different actors within the boundary [61]. We will administer different types of structured and pre-validated (through pilot testing) questionnaires. The first one will include aspects of demographic information, knowledge of the system, their interaction within the category and beyond the category, and factors driving for the interaction, for the managerial and provider level actors. The network questionnaire aims to collect the frequency of contact and level of collaboration within the own system as well as with the other system [57, 59]. Collaboration will be assessed with a scale adapted from established network analytic methods [62]. Participants will be asked to select the response that best describes the current relationship with each of the actors from different levels. In addition to this, some specific details for different actors will also be collected. The second questionnaire, which will be administered to the community households, contains some demographic details, socioeconomic information, animal handling practices, attitude towards preventive practices, and contact and experiences with both the human and animal health system during and after the outbreak and during non-outbreak periods.

To assess the current convergence points of the human and animal health system actors with their strengths we will adapt network analysis for the network data. Social network analysis provides insights into stakeholder relationships, especially the dynamics within a health system [60]. Social network analysis is defined as a distinctive set of methods used for mapping, measuring and analysing the social relationships between people, groups and organisations [63, 64]. As social network analysis has proved that it can be used to help understand the nature of relations between actors within a system and how these relationships influence the structure of a system [64, 65]. A visualisation of the current interactions and quantified outcomes, such as betweenness, centrality, density, distance and reachability, will be the result of this analysis. UCINET version 6 [66] will be used for this analysis.

### Method for objective 4

Development of a convergence strategy is an iterative process exploring the best possible options for establishing horizontal collaboration between two vertical systems. In this phase, we attempt to document how convergence between the two systems could be strengthened through a vignette approach. The Vignette technique is a qualitative approach that documents the decision-making and possible convergence pattern between actors of two systems. The Vignette technique can elicit perceptions, opinions, beliefs and attitudes from responses or comments to stories depicting scenarios and situations [67]. Vignette methods are being used not only in clinical settings [68] for decision-making, but also in public health settings [69] to solve complex issues. A semi-structured Vignette questionnaire hypothesising the ideal convergence and collaborative actions amongst the health system actors will be administered to the sampled stakeholders through face-to-face interviews. Thus, we will gather as many convergence strategies as possible through interviews and then validate these strategies to ensure their feasibility. This validation will be done through the policy Delphi technique with health system experts. The Delphi methodology was developed at the RAND Corporation in the 1950s in order to make more reliable forecasts of the future [70]. Though certain basic principles of procedure and selection are the same, this technique has considerably changed its applications and objectives until now. The key difference of the traditional Delphi method is that the objective is not to develop consensus but to identify the widest possible range of valid options/solutions to a policy problem [71, 72].

The sample for this objective will be recruited from the managerial level and providers’ level. All actors who will not yet have been interviewed will be sampled based on purposive sampling. Initially, 10–12 actors from each level will be interviewed; subsequently, we will proceed in recruiting new subjects until a certain saturation of responses is reached. For the policy Delphi survey, additional samples, i.e. experts from the academia, research, government, international/national agencies, etc., who have experience in policy formulation will be approached purposively. These experts are not necessarily from the study area. We will approach national policy-makers, national health mission, health policy and planning division, academia from the field of infectious diseases and veterinary science, national nodal persons from surveillance agencies, etc. We will send all documented options of potential horizontal collaboration to these experts and will seek the opinions and feedback through an online survey. We will use Survey Monkey software [73] to develop the online survey and invite potential health system experts via email. Participants will be asked to rank the importance of items in the grid by rating each item on a Likert rating scale (1–10; 1 – strongly disagree, 10 – strongly agree). They also will be asked to provide recommendations regarding any addition and/or deletions to the list of proposed items and for any other comments/suggestions. Each survey will take 15–25 min to complete, with the option to complete it over several sessions and to allow participants to review their answers prior to final submission. In case of high non-response, the investigator will personally approach these experts to document their responses through face-to-face interaction.

Vignette data will be handled like other qualitative data and will be reported using the Consolidated Criteria for Reporting Qualitative Research [51], after analysis through ATLAS.ti version 8 [52]. The Policy Delphi responses will be in quantitative form as collected with a Likert scale as well as qualitative statements consisting of feedback, suggestions and comments. Therefore, de-identified results comprising overall scores for each item (analysed in a number of ways, e.g. percentage, mean, median, SD, range and proportions for the quantitative data and thematic analysis for the qualitative data) and narrative summary of findings, comments and suggestions will be obtained. Although most research recommends having a consensual mean score of at least 7 out of 10 in the Delphi survey to be included for further consideration, at this point we are not fixing any strategy for the same. After obtaining all responses, we will decide the consensual mean score cut off for inclusion criteria. Finally, a ranking of item importance will be made to rationalise the number of items and model this according to the CONSORT statement and TIDieR checklist for consistency [74, 75]. Final options from this survey outcome will be considered to develop a system convergence model and will be presented through a graphical system figure.

### Method for objective 5

To address this objective, participatory stakeholder workshops are planned to capture the factors essential for convergence. This participatory method is well established in public health research for various purposes [76]; herein, we will employ it to capture the factors that play a role between the health systems to develop a convergence.

Approximately 10–12 actors, who have previously attended the prioritisation workshop from the managerial and the providers’ level, will be recruited for this objective. The workshop will provide the most important input for the analysis. It is highly important that all stakeholder groups are adequately represented and get an equal voice during this process. During the workshop, all stakeholders will be briefed about the aim of the workshop and will be presented with the findings of the previous objectives. The workshop will consist of three phases, as described below.

- ■ *Phase I:* Describing the system (system image, system problems), setting up the variables of interplay (acquisition of hard or soft variables with a description) and criteria matrix (check the representativeness of variables from a system viewpoint)
- ■ *Phase II:* Consensus effect matrix (define and assess variable interlinkages) with the role of variables (evaluate and systematic role allocation of variables)
- ■ *Phase III:* Cause–effect system (visual representation of variable linkages) with system model (selecting and analysing relevant feedback loops)

To begin the brainstorming and listing of the health system factors, these elements need to be categorised into aggregate variables. The Sensitivity Model^®^ [77] provides a tool (Criteria Matrix) to ensure the variable set is representative of the system. It should be noted that the Sensitivity Model^®^ is not set up linearly, so that the choice of variables and their definitions can be altered during any stage of the process. Ultimately, a set of 20 to 30 variables influencing convergence, such as human resources, common budget, knowledge about zoonoses, etc., should be defined. Information from the brainstorming can flow into variables as qualitative inputs; additionally, both quantitative and qualitative data is entered during discussion. During the next stage of the workshop, the participant group will be divided into 3–4 sub-groups. Each sub-group needs to complete the Cross-Impact-Matrix of the Sensitivity Model^®^, where the strength of impact between the various system variables is determined. The results of the sub-groups are then discussed and a consensus Cross-Impact-Matrix is created. During this stage, some variables may be redefined to ensure consensus. The Sensitivity Model^®^ utilises the data from the Cross-Impact-Matrix to determine the systemic role of each system variable. The next workshop stage requires the development of the Effect System, which is similar to the Cross-Impact-Matrix but does not focus on the strength of impact but the direction. This step is highly important, as the Effect System forms the basis for the identification of the regulating feedback system. The Sensitivity Model^®^ provides a tool to visualise the relationships between the various variables and aids with the analysis of the feedback system. The resulting Effect System forms a key output and enables the identification of important and less important system variables. The Effect System also indicates the viability and self-regulation of the system and thus is crucial for testing all the possible convergence options.

After the workshop phase, data collection will be completed. The model developed during the workshop will be used to test the various hypotheses. Initially, the viability and sustainability of the system is analysed through the eight basic bio-cybernetic principals. The number of feedback loops, as well as the dominance of negative feedback over positive feedback are important indicators for the viability of the system. The role of health system convergence can be determined through various simulations.

The analysis of the workshop will use the computerised Sensitivity Model^®^ developed by Vester [77], which has its foundation in cybernetics and is designed to guide stakeholders to visualise and analyse the dynamics of complex systems. Through various policy simulation tests, the outcome of this participatory workshop and the simultaneous analysis will provide a comprehensive and visual description of the variable interactions in the convergence of the health systems.

## Expected outcomes

The expected outcome from this study will be a system model for describing and enhancing convergence between the human and the animal health system, based on the factors that affect the convergence process for effective prevention and control of zoonotic diseases in Ahmedabad, India. This will provide an insight into the entry points for One Health thinking (exploring the points for horizontal linking) within the complex (public as well as private) health system at a city level.

As far as we can see, this will be the first study of its kind to understand the health system from a One Health perspective in an Indian city. With the synchronised objectives of this study, it will not only document the current degree of interaction between One Health stakeholders, but also develop a convergence model for the human and the animal health systems, which will facilitate the One Health approach at city level. Recommendations from this study could be a potential source for future One Health policy and planning.
