# Correction to: Convergence model for effectual prevention and control of zoonotic diseases: a health system study on ‘One Health’ approach in Ahmedabad, India

**DOI:** 10.1186/s12961-019-0481-7

**Published:** 2019-08-09

**Authors:** Sandul Yasobant, Walter Bruchhausen, Deepak Saxena, Timo Falkenberg

**Affiliations:** 1 Center for Development Research (ZEF), University of Bonn, Bonn, Germany; 2 Faculty of Medicine, University of Bonn, Bonn, Germany; 3 Institute of History and Ethics of Medicine, University of Cologne, Faculty of Medicine and University Hospital Cologne, Cologne, Germany; 4 Indian Institute of Public Health Gandhinagar (IIPHG), Gujarat, India; 5 GeoHealth Centre, Institute for Hygiene and Public Health, University of Bonn, Bonn, Germany

**Correction to: Health Res Policy and Syst (2018) 16:124**

**https://doi.org/10.1186/s12961-018-0398-6**

It was highlighted that the original article [1] contained an error in the Methods section, specifically in Study Section. The number urban health centres should be 72 instead of 6. This Correction article shows the incorrect and correct statement in the Methods section.

**Incorrect statement:**

The rural areas of Ahmedabad have one district hospital, six community health offices and 36 primary health centres [3], whereas the urban areas of Ahmedabad have six urban health centres, six medical colleges and one homeopathy college as well as being well facilitated by private companies for human health [2].

**Correct statement:**

The rural areas of Ahmedabad have one district hospital, six community health offices and 36 primary health centres [3], whereas the urban areas of Ahmedabad have 72 urban health centres, six medical colleges and one homeopathy college as well as being well facilitated by private companies for human health [2].
